# The Relationship between Sport-Related Concussion and Sensation-Seeking

**DOI:** 10.3390/ijms21239097

**Published:** 2020-11-30

**Authors:** Spencer W. Liebel, Kathryn L. Van Pelt, Gian-Gabriel P. Garcia, Lauren L. Czerniak, Michael A. McCrea, Thomas W. McAllister, Steven P. Broglio, on behalf of the CARE Consortium Investigators

**Affiliations:** 1 Department of Psychiatry, University of Michigan, Ann Arbor, MI 48109, USA; 2 Michigan Concussion Center, University of Michigan, Ann Arbor, MI 48109, USA; broglio@umich.edu; 3 Sanders-Brown Center on Aging, University of Kentucky, Lexington, KY 40504, USA; kathryn.oconnor@uky.edu; 4 Department of Industrial and Operations Engineering, University of Michigan, Ann Arbor, MI 48109, USA; garciagg@umich.edu (G.-G.P.G.); czernl@umich.edu (L.L.C.); 5 Department of Neurosurgery, Medical College of Wisconsin, Wauwatosa, WI 53226, USA; mmccrea@mcw.edu; 6 Department of Psychiatry, Indiana University School of Medicine, Indianapolis, IN 46202, USA; twmcalli@iupui.edu

**Keywords:** concussion, sensation-seeking, concussion management and care, college athletes

## Abstract

Sensation-seeking, or the need for novel and exciting experiences, is thought to play a role in sport-related concussion (SRC), yet much remains unknown regarding these relationships and, more importantly, how sensation-seeking influences SRC risk. The current study assessed sensation-seeking, sport contact level, and SRC history and incidence in a large sample of NCAA collegiate athletes. Data included a full study sample of 22,374 baseline evaluations and a sub-sample of 2037 incident SRC. Independent samples *t*-test, analysis of covariance, and hierarchical logistic regression were constructed to address study hypotheses. Results showed that (1) among participants without SRC, sensation-seeking scores were higher in athletes playing contact sports compared to those playing limited- or non-contact sports (*p* < 0.001, *R^2^* = 0.007, η^2^_p_ = 0.003); (2) in the full study sample, a one-point increase in sensation-seeking scores resulted in a 21% greater risk of prior SRC (OR = 1.212; 95% CI: 1.154–1.272), and in the incident SRC sub-sample, a 28% greater risk of prior SRC (OR = 1.278; 95% CI: 1.104–1.480); (3) a one-point increase in sensation-seeking scores resulted in a 12% greater risk of incident SRC among the full study sample; and (4) sensation-seeking did not vary as a function of incident SRC (*p* = 0.281, η^2^_p_ = 0.000). Our findings demonstrate the potential usefulness of considering sensation-seeking in SRC management.

## 1. Introduction

Mild traumatic brain injury (mTBI), or concussion, has been recognized as a major public health problem. Approximately 1.6–3.8 million concussions occur annually in the United States during organized or recreational sports [1]. Sport-related concussion (SRC) causes symptoms such as headache, nausea, and sensitivity to light and noise as well as impairments in cognition, balance, and other areas of function [2,3,4]. The number, frequency, and severity of these symptoms vary significantly across and within athletes such that there does not yet exist reliable post-SRC symptom profiles. Contributing to this heterogeneity is the fact that, while most athletes achieve SRC-related symptom resolution within days or weeks, a significant minority experience such symptoms far beyond this timeframe [5]. These myriad variables in SRC management emphasize the need for identification of SRC risk factors as a starting point for SRC prevention.

Personality traits have been suggested as one such possible avenue for SRC risk factor identification [6]. Impulsivity is a common neurobehavioral feature following traumatic brain injury and there exists evidence suggestive of a specific relationship between higher impulsivity and SRC [7,8,9,10,11,12]. Kerr and colleagues [13] reported that former collegiate athletes with a history of two or more SRC had higher mean impulsivity scores on the Barratt Impulsiveness Scale-15 [14] than athletes reporting no history of SRC. No such relationship was found for athletes reporting one prior SRC. A separate study found that the caregivers of children and adolescents who had sustained an mTBI reported increased impulsivity and impatience in the mTBI patients [15]. Related to impulsivity, sensation-seeking, as defined by Zuckerman [16], “is a trait defined by the seeking of varied, novel, complex, and intense sensations and experiences, and the willingness to take physical, social, legal, and financial risks for the sake of such experiences” (p. 27) and is thought to play a role in SRC. Evidence from multitrait-multimethod analysis and meta-analysis show high discriminant validity among measures of sensation-seeking and impulsive traits and confirm that they comprise distinct factors [17,18]. Thus, while previous research has found changes in impulsivity following SRC, it remains unknown how sensation-seeking relates to SRC, and more importantly, how it influences SRC risk.

While research on the relationships between SRC and sensation-seeking is relatively limited, there are reports that such a relationship exists. A study by Dretsch and colleagues [19] reported that military service members with prior SRC reported higher scores on impulsivity and sensation-seeking than those with no prior SRC. Along similar lines, nonprofessional male rugby players who reported high sensation-seeking possessed a significantly higher SRC incidence rate compared to those reporting low sensation-seeking [20]. Thus, some prior research has demonstrated that sensation-seeking is associated with SRC [21,22,23]. Despite these findings, methodological considerations, including the lack of prospective design and geographically restricted samples, limit generalizability.

Sensation-seeking may also contribute to an athlete’s choice of which sport(s) to pursue. Indeed, it seems reasonable that the desire to experience novel and/or exciting stimuli may predispose an athlete to participate in sports that involve greater physical contact and risk, like American football, rather than sports with less or no contact and less risk [24]. Research has shown that certain personality traits (i.e., neuroticism, extraversion, and impulsivity) are associated with risk-taking behaviors [6,25,26], yet the association of personality traits with SRC is unclear [27,28,29,30]. To our knowledge, no prior studies have examined the relationships between sensation-seeking and sport contact level explicitly.

With these details in mind, the purpose of the current study was to further investigate the relationship between SRC history and sensation-seeking. We hypothesized that (1) collegiate athletes with higher sensation-seeking will be disproportionally represented in contact sports relative to limited or non-contact sport athletes; (2) SRC history will be associated with sensation-seeking after controlling for sex differences and sport contact level; (3) incident SRC during the study period will be associated with self-report of sensation-seeking, while controlling for sex differences, sport contact level, and the number of years played; and (4) self-reported sensation-seeking will vary as a function of incident SRC after controlling for sex differences, SRC history, and sport contact level.

## 2. Results

BSSS-8 scores for the overall sample and the incident SRC sub-sample are presented in Table 1. Analyses showed that in the full study sample, male participants reported significantly higher BSSS-8 scores than did female participants (*t*(18901) = 9.162, *p* < 0.001, *d* = 0.142). In the incident SRC sub-sample, male participants reported significantly higher BSSS-8 scores than did female participants (*t*(1586) = 2.022, *p* = 0.043, *d* = 0.099).

The mean BSSS-8 score for the overall study sample (*M* = 3.08, SD = 0.71; *n* = 22,374) was significantly lower (*p* < 0.001) than the reported mean BSSS-8 score of the measure’s normative sample (*M* = 3.74, SD = 0.71; *n* = 1263) [30].

### 2.1. Hypothesis 1

In the full study sample, ANCOVA results showed a significant difference between sport contact level and BSSS-8 scores while adjusting for sex (*p* < 0.001, *R^2^* = 0.007, η^2^_p_ = 0.003). Post hoc tests using a Bonferroni adjustment showed there were significant BSSS-8 mean differences between all sport contact levels: contact versus limited-contact (*p* = 0.017), contact versus non-contact (*p* < 0.001), and limited versus non-contact (*p* = 0.023). In the incident SRC sub-sample, there was no difference in BSSS-8 scores by sport contact level while adjusting for sex differences (*p* = 0.777, *R^2^* = 0.001, η^2^_p_ = 0.000).

### 2.2. Hypothesis 2

In the full study sample, ANCOVA results showed significant mean differences of BSSS-8 scores by the number of prior SRC after controlling for sex differences and sport contact level (*p* < 0.001, *R^2^* = 0.010, η^2^_p_ = 0.004). Post hoc tests using a Bonferroni adjustment showed that participants with one (*p* < 0.001) and two (*p* < 0.001) prior SRC had higher BSSS-8 scores than those with zero prior SRC. Participants with two prior SRC had higher BSSS-8 scores than those with one prior SRC (*p* < 0.001). Participants with three or more prior SRC did not have different BSSS-8 scores than any other prior SRC level. In the incident SRC sub-sample, there was also a significant mean difference between BSSS-8 scores by the number of prior SRC after controlling for sex differences and sport contact level, (*p* = 0.004, *R^2^* = 0.010, η^2^_p_ = 0.012). Post hoc tests using a Bonferroni adjustment showed that participants with one (*p* = 0.028) prior SRC had higher BSSS-8 scores than those reporting zero prior SRC. There were no other significant differences in mean BSSS-8 scores at any prior SRC level.

A hierarchical logistic regression model constructed in the full study sample found that an increase of 0.125 in BSSS-8 total scores (or a one-point increase on any item of the BSSS-8) resulted in a 21% greater risk of prior SRC (OR = 1.212; 95% CI: 1.154–1.272) (Table 2a) after accounting for participant sex and sport contact level. In a separate hierarchical logistic regression model among the incident SRC sub-sample, participants had a 28% greater risk for incident SRC with every one-point increase on any single BSSS-8 item (OR = 1.278; 95% CI: 1.104–1.480) (Table 2b) after accounting for participant sex and sport contact level.

### 2.3. Hypothesis 3

After accounting for participant sex, sport contact level, and the number of years played, a one-point increase on any single BSSS-8 item resulted in a 12% greater risk of incident SRC among the full study sample (OR = 1.119; 95% CI: 1.039–1.204) (Table 2c).

### 2.4. Hypothesis 4

Results of repeated measures ANCOVA, controlling for the effects of participant sex, SRC history, and sport contact level, showed that participant BSSS-8 scores did not vary as a function of incident SRC, *p* = 0.281, η^2^_p_ = 0.000. However, these analyses showed that participant sex accounted for significant mean BSSS-8 score differences at participants’ first and second evaluation, without incident SRC, *p* < 0.001, η^2^_p_ = 0.038. Indeed, males (*n* = 4442) reported higher mean BSSS-8 scores at their first (*M* = 3.33 (*SD* = 0.67)) and second evaluations (*M* = 3.36 (*SD* = 0.69)) than did females (*n* = 2196) at their first (*M* = 3.08 (*SD* = 0.67)) and second (*M* = 3.07 (*SD* = 0.71)) evaluations.

## 3. Discussion

The current study investigated the relationship between sensation-seeking and SRC in a large sample of college-age athletes from NCAA institutions in the United States. In addition to what is known about the biomechanical causes of SRC, it is important to elucidate behavioral and personal factors that influence risk of head injury in sports. Overall, the current study found that participants who played contact sports reported higher sensation-seeking than those who played limited or non-contact sports. Further, sensation-seeking scores were positively associated with SRC history and predicted incident SRC. Last, incident SRC did not appear to alter sensation-seeking scores, suggesting a more stable trait among athletes.

Among the full study sample, our analyses found that male participants reported significantly higher sensation-seeking than did female participants, consistent with prior research [31,32,33]. The same pattern of higher sensation-seeking among males compared to females was also found in the incident SRC sub-sample. To our knowledge, this is the first study to report such sex differences in sensation-seeking ratings in collegiate athletes. Thus, these findings support and meaningfully extend prior research and provide additional data regarding these behaviors which have significant physical, psychological, social, and academic correlates [34]. Of note, the current study’s full sample reported lower mean sensation-seeking scores than the reported mean scores of the BSSS-8 normative sample [35]. It is likely that differences in sample sizes, group demographic variables, and activity levels (e.g., middle and high school vs. collegiate) account for these differences. Importantly, it is likely the inhibitory control among the CARE Consortium participants is more developed than those of the adolescent BSSS-8 normative sample due to differences in brain morphology and development. A comparable collegiate athlete sample, however, is not available in the published literature.

In the first of our primary hypotheses, we posited that collegiate athletes with higher sensation-seeking would be disproportionally represented in contact sports relative to limited or non-contact sport athletes. Results partially supported this hypothesis. Among the full study sample, sensation-seeking scores were higher in athletes playing contact sports compared to limited or non-contact sports. In fact, athletes participating in greater levels of sport contact reported higher sensation-seeking than those playing a sport with less or no contact. While there is no prior research investigating these factors in a sample of NCAA collegiate athletes, there exists some evidence that suggests that individuals who participate in high-risk sports, analogous to contact sports in the current study, report higher sensation-seeking than those who play low-risk sports [6,36]. However, in the incident SRC sub-sample, no differences in sensation-seeking scores were found across sport contact levels. As we statistically controlled for the effects of participant sex in these analyses (a factor previous analyses from the current study demonstrated to be significantly related to sensation-seeking reporting), it appears the preference for or disposition towards sports of varying contact levels is not influenced by sensation-seeking among collegiate athletes’ who sustain a SRC. This is somewhat incongruous with prior research that reports athletes with particular personality traits, including impulsivity and high sensation-seeking, may prefer high-risk or contact sports [6,37]. An alternative explanation for these findings is that athletes with incident SRC, even among those participating in low-contact sports, may already possess “high” sensation-seeking which could potentially explain why they sustained an incident SRC in the first place.

The second hypothesis of the current study, that SRC history would be associated with sensation-seeking, was also generally supported. In both the full study sample and incident SRC sub-sample, sensation-seeking significantly increased the risk of sustaining a prior SRC. That is, a one-point increase on any given sensation-seeking score question at baseline in the full study sample increased participants’ risk by 21% of having a prior SRC and increased risk by 28% in the incident SRC sub-sample. These findings demonstrate the utility of assessing sensation-seeking when predicting prior SRC and provides evidence that these measurements help inform which athletes possess a greater likelihood of having sustained a SRC.

Sensation-seeking ratings were an independent risk factor for incident SRC after controlling for sex, sport contact level, and the number of years played, supporting our third hypothesis. Indeed, a one-point increase in sensation-seeking scores at baseline was associated with a 12% greater likelihood of sustaining an incident SRC after controlling for the other variables in the model. This finding suggests that sensation-seeking exerts a meaningful influence on college athletes’ propensity to sustain an SRC. One of the key goals of SRC research is to find predictors and determinants of SRC as means to better manage and care for athletes. These findings demonstrate the utility of measuring sensation-seeking by athletic trainers, neuropsychologists, and medical staff to identify those at highest injury risk and potentially intervene prior to injury, though clinical effects may be small. For example, when included as a part of athletes’ pre-season baseline SRC management examination, relevant clinicians could provide additional and/or more in-depth SRC psychoeducation to those athletes with higher self-reported sensation-seeking as a means to potentially reduce the likelihood of those athletes sustaining a future SRC. Further, for those athletes self-reporting clinically significant current sensation-seeking tendencies, a referral to a licensed clinical psychologist or other licensed psychotherapist may be considered to assist in better managing and ameliorating the effects of sensation-seeking through psychotherapeutic intervention(s). Our results also may suggest that measures of sensation-seeking could be used in resource-limited settings to guide the decisions of which athletes receive baseline testing and which do not.

The fourth hypothesis of our study, that sensation-seeking would vary as a function of incident SRC, was not supported. That is, incident SRC did not change post-SRC sensation-seeking scores. Such stability in BSSS-8 scores in this sample suggests that sensation-seeking is a trait—rather than state-level personality factor, consistent with previous research [38,39]. Thus, repeating sensation-seeking assessment via the BSSS-8 after an athlete’s baseline evaluation may have limited clinical utility, especially when considering the additional time and effort required to collect these data.

The current study excluded from analyses those participants with ADHD due to possible overlap of symptoms of that disorder and sensation-seeking. At a minimum, sensation-seeking is conceptually related to symptoms of ADHD. Extant research on the relationship between SRC and ADHD is mixed, but there exists evidence that ADHD may confer increased risk for SRC [40,41,42]. It remains unclear, however, if the increased risk is related to a relative inability to maintain attention, more frequent impulsive decision making, or some other factor related to the condition. More research is needed to appropriately elucidate these relationships and how, if at all, they relate to sensation-seeking.

### Limitations

The current study should be considered in light of several limitations. First, it is possible that a proportion of those study participants who self-reported receiving a diagnosis of ADHD did not in fact have that disorder or received an inaccurate diagnosis in place of another mental health concern. Although comprehensive neuropsychological evaluations were not implemented in the study design, a multi-informant, multi-method approach would ensure the accuracy of participants’ ADHD history. Conversely, it is possible that some study participants with a diagnosis of ADHD did not report their disorder, which may have also introduced unintended variance into our analyses. Similarly, study participants self-reported the number of SRC they had sustained prior to their participation and may have introduced error via potential over- or underestimation. Third, the reliability and validity of the BSSS-8, the present study’s measure of sensation-seeking, have not been evaluated in college-age student athletes. While much of the research that uses the BSSS-8, including its normative sample, has been conducted in adolescents similar in age to that of the participants’ of the current study, there exists the possibility of structural and functional brain maturation differences between those groups and those of the current study. More research, including formally establishing the validity of the BSSS-8 for use in collegiate student athletes is needed to address this issue. Fourth, BSSS-8 total scores were used in analyses rather than constituent subscales (e.g., thrill and adventure seeking), eliminating possible meaningful variance within participants’ scores. Fifth, it is possible that the incident SRC sub-sample did not include all participants who sustained an SRC during study participation due to nondisclosure, likely leading to an underestimation of the association between sensation-seeking scores and SRC [43,44]. Lastly, the estimated effect sizes for the statistically significant relationships described above were small. It is likely our large sample sizes, as well as possible mediating and moderating variables (e.g., cognitive functioning) that we did not consider, contributed to these significant findings. Such small effects sizes limit the generalizability of findings and call into question their clinical or “real world” utility.

## 4. Material and Methods

### 4.1. Participants

Data for this study were collected by the Concussion Assessment, Research, and Education (CARE) Consortium [45]. Participants in the current study consisted of individuals from 30 (29 currently contribute data) National Collegiate Athletic Association (NCAA) universities in the United States who participated in an NCAA sport during the 2014–2018 academic years. The dataset included 34,561 pre-season baseline examinations where, among other variables, participants self-reported the number of SRC prior to study involvement [46]. All participants sustaining a SRC during the study period underwent additional examinations by the local medical team at their institution using a standardized injury definition [47]. From this overall sample, we identified an incident SRC sub-sample in order to investigate hypotheses. Participants who self-reported a diagnosis of attention-deficit/hyperactivity disorder (ADHD; *n* = 1830) or for whom ADHD status data were missing (*n* = 489) were excluded from analyses due to the potential overlap of symptoms of ADHD and sensation-seeking. As the intent of this investigation was to focus on SRC, service academy cadets that did not participate in a varsity sport (*n* = 9868) were excluded from our analysis. The final sample included 22,374 baseline evaluations and 2037 incident SRC, referred to hereafter as the full study sample and incident SRC sub-sample, respectively. Table 3 provides participant demographic information. All participants provided written informed consent which was approved by the local institutional review board (University of Michigan Institutional Review Boards (IRBMED), approval: HUM00093221, 6/11/2014) and US Army Human Research Protection Office.

### 4.2. Measures

Sensation-seeking was assessed at the time of enrollment as measured via the Brief Sensation Seeking Scale-8 (BSSS-8), an identified National Institutes of Health Common Data Element for SRC [35,48,49]. For participants in the incident SRC sub-sample, they were again administered the BSSS-8 annually as part of their preseason baseline evaluation. The BSSS-8 conceptualizes sensation-seeking into four components, constituted by two scale items each: thrill and adventure seeking, experience seeking, disinhibition, and boredom susceptibility. The psychometric reliability and validity of the BSSS-8 is adequate [35]. BSSS-8 responses were indicated on five-point scales (“strongly disagree” to “strongly agree”). The present study used the BSSS-8 total score in analyses, which is obtained by averaging together participants’ responses on all eight items, in accordance with prior research [48,50,51]. The minimum and maximum BSSS-8 scores were 1 and 5, respectively. In order to aid in the accurate interpretation of results, we provide the relevant incremental value of a 1-point change on any of the eight BSSS-8 items (1/8 or 0.125) rather than reporting how a 1-point change on all items of the BSSS-8 affects findings. In the full study sample, the BSSS-8 demonstrated a Cronbach’s alpha of 0.795. In the incident SRC sub-sample, Cronbach’s alpha for the BSSS-8 was 0.787.

The contact level of the sport each participant played was classified according to prior research [45,49]. Classifications included “contact” (e.g., American football), “limited contact” (e.g., baseball), and “non-contact” (e.g., golf). For hypothesis 3, the number of years the participant reported having played their sport was entered as a covariate in analyses in order to control for possible exposure effects. Participants who reported playing their sport prior to the age of 4 or reported playing zero years prior to study participation were excluded from these analyses in accordance with prior research [52].

### 4.3. Statistical Analysis

Independent samples *t*-tests comparing male and female participants’ BSSS-8 scores were conducted. In order to investigate whether BSSS-8 scores (dependent variable) varied by sport contact level (independent variable) after controlling for the effects of participant sex and sport contact level, analysis of covariance (ANCOVA) were constructed. Hierarchical logistical regression models predicting SRC history and incident SRC by BSSS-8 scores were also constructed. Finally, repeated measures ANCOVA were constructed to investigate whether BSSS-8 scores varied as a function of incident SRC after controlling for sex differences, SRC history, and sport contact level. Appropriate effect size metrics were calculated. Graphical analysis of BSSS-8 scores indicated these data were normally distributed. The significance threshold for all analyses was set at *p* < 0.05, two tailed. Statistical Package for Social Sciences (SPSS 26.0 for Windows, SPSS, Chicago, IL, USA) was used for all analyses.

## 5. Conclusions

Our study is the first of its kind to prospectively examine the relationship between sensation-seeking and SRC history and incidence. Consistent with our hypotheses, we found that males reported higher sensation-seeking than females and higher sensation-seeking scores were reported by those athletes playing contact sports compared to those playing low- or non-contact sports. Our results also showed an association between sensation-seeking and history and incidence of SRC. Our findings demonstrate the potential usefulness of considering behavioral and personality factors, including sensation-seeking, in SRC management but clinical effects may be small. Given the complexity of SRC injury and the severity of associated consequences, it is imperative to improve our understanding of the factors (e.g., sensation-seeking) that may potentially predispose athletes to increased SRC risk. Future research should continue to focus on these important factors that assist in SRC prediction.

## Figures and Tables

**Table 1:** Participant BSSS-8 scores for each demographic category.

	Full Study Sample	Incident SRC Sub-Sample
	(*n* = 22,374)	(*n* = 2037)
Overall	3.08 (0.71)	3.12 (0.71)
Male	3.12 (0.72) *	3.15 (0.71) *
Female	3.02 (0.69) *	3.08 (0.71) *
Sport contact level:		
Contact sport	3.07 (0.72)	3.12 (0.72)
Limited contact sport	3.07 (0.69)	3.10 (0.68)
Non-contact sport	3.09 (0.70)	3.12 (0.70)
Prior concussions:		
0	3.06 (0.71)	3.07 (0.72)
1	3.13 (0.71)	3.20 (0.69)
2	3.18 (0.68)	3.16 (0.71)
3	3.19 (0.68)	3.19 (0.69)
≥4	3.33 (0.77)	3.12 (0.71)

Note. Values presented as Mean (Standard Deviation). * Denotes significant between-groups mean differences.

**Table 2:** (**a**) Hierarchical logistic regression model of sensation-seeking behavior scores predicting prior SRC history in the full study sample. (**b**) Hierarchical logistic regression model of sensation-seeking behavior scores predicting prior SRC history in the incident SRC sub-sample. (**c**) Hierarchical logistic regression model of sensation-seeking behavior scores predicting incident SRC in the full study sample.

(a)	*B*	SE (*B*)	Wald	OR	CI (95%)
Step 1					
Participant Sex	0.136	0.037	13.405 ***	1.145	1.065, 1.232
Sport Contact Level	−0.545	0.025	480.102 ***	0.580	0.553, 0.609
Step 2					
Participant Sex	0.156	0.37	17.535 ***	1.168	1.086, 1257
Sport Contact Level	−0.552	0.025	491.925 ***	0.576	0.548, 0.605
BSSS-8 Total Scores	0.192	0.025	59.660 ***	1.212	1.154, 1.272
(**b**)	***B***	**SE (*B*)**	**Wald**	**OR**	**CI (95%)**
Step 1					
Participant Sex	0.182	0.115	2.491	1.199	0.957, 1.502
Sport Contact Level	−0.445	0.088	25.799 ***	0.641	0.540, 0.761
Step 2					
Participant Sex	0.201	0.115	3.040	1.223	0.975, 1.534
Sport Contact Level	−0.451	0.088	26.481 ***	0.637	0.536, 0.756
BSSS-8 Total Scores	0.245	0.075	10.733 ***	1.278	1.104, 1.480
(**c**)	***B***	**SE (*B*)**	**Wald**	**OR**	**CI (95%)**
Step 1					
Participant Sex	0.215	0.056	14.477 ***	1.239	1.110, 1.384
Sport Contact Level	−0.643	0.042	235.066 ***	0.526	0.484, 0.571
Years Played	0.007	0.007	0.952	1.007	0.993, 1.020
Step 2					
Participant Sex	0.225	0.056	15.829 ***	1.252	1.121, 1.398
Sport Contact Level	−0.646	0.042	237.160 ***	0.524	0.483, 0.569
Years Played	0.007	0.007	1.003	1.007	0.994, 1.020
BSSS-8 Total Scores	0.112	0.038	8.845 **	1.119	1.039, 1.204

*Note.* ** *p* < 0.01, *** *p* < 0.001. BSSS-8 = Brief Sensation Seeking Scale-8; Years Played = number of years participants reported playing their sport; OR = odds ratio.

**Table 3:** Descriptive statistics of participants at first baseline evaluation.

	Full Study Sample (*n* = 22,374)		Incident SRC Sub-Sample (*n* = 2037)	
	*n*	Percent	*n*	Percent
Male	12,610	56.40%	1203	59.10%
Female	9763	43.60%	834	40.90%
Age, *M* (SD)	19.12 (1.37)		18.94 (1.21)	
Sport contact level:				
Contact sport	10,607	47.40%	1394	68.40%
Limited contact sport	6861	30.70%	434	21.30%
Non-contact sport	4906	21.90%	209	10.30%
Prior concussions:				
0	16,751	74.90%	1195	58.70%
1	4106	18.40%	582	28.60%
2	983	4.40%	158	7.80%
3	277	1.20%	61	3.00%
≥4	110	~0.50%	28	~1.30%
Unknown	148	0.70%	13	0.60%

Note. Prior concussions = number of self-reported concussions sustained prior to study participation.

## Abbreviations

SRC	Sport-Related Concussion
BSSS-8	Brief Sensation Seeking Scale-8
mTBI	Mild Traumatic Brain Injury
ADHD	Attention-Deficit/Hyperactivity Disorder
ANCOVA	Analysis of Covariance
NCAA	National Collegiate Athletic Association
CARE	Concussion, Assessment, Research, and Education Consortium

## References

[1] Langlois J.A., Rutland-Brown W., Wald M.M. (2006). The epidemiology and impact of traumatic brain injury: A brief overview. J. Head Trauma Rehabil..

[2] McAllister T., McCrea M. (2017). Long-term cognitive and neuropsychiatric consequences of repetitive concussion and head-impact exposure. J. Athl. Train..

[3] Rabinowitz A.R., Levin H.S. (2014). Cognitive sequelae of traumatic brain injury. Psychiatr. Clin. N. Am..

[4] Wood T.A., Hsieh K.L., An R., Ballard R.A., Sosnoff J.J. (2019). Balance and gait alterations observed more than 2 weeks after concussion: A systematic review and meta-analysis. Am. J. Phys. Med. Rehabil..

[5] Broglio S.P., Hainline B., Stern R.A. (2018). Return to play following sports-related concussion. Handbook of Clinical Neurology, Sports Neurology.

[6] McEwan D., Boudreau P., Curran T., Rhodes R.E. (2019). Personality traits of high-risk sport participants: A meta-analysis. J. Res. Personal..

[7] Bigler E.D. (2007). Anterior and middle cranial fossa in traumatic brain injury: Relevant neuroanatomy and neuropathology in the study of neuropsychological outcome. Neuropsychology.

[8] Goswami R., Dufort P., Tartaglia M.C., Green R.E., Crawley A., Tator C.H., Wennberg R., Mikulis D.J., Keightley M., Davis K.D. (2016). Frontotemporal correlates of impulsivity and machine learning in retired professional athletes with a history multiple concussions. Brain Struct. Funct..

[9] Greve K.W., Sherwin E., Stanford M.S., Mathias C., Love J., Ramzinski P. (2001). Personality and neurocognitive correlates of impulsive aggression in long-term survivors of severe traumatic brain injury. Brain Inj..

[10] Kocka A., Gagnon J. (2014). Definition of impulsivity and related terms following traumatic brain injury: A review of the different concepts and measures used to assess impulsivity, disinhibition and other related concepts. Behav. Sci..

[11] Mosti C., Coccaro E.F. (2018). Mild traumatic brain injury and aggression, impulsivity and history of other- and self-directed aggression. J. Neuropsychiatry Clin. Neurosci..

[12] Rochat L., Ammann J., Mayer E., Annoni J.-M., Van der Linden M. (2009). Executive disorders and perceived socio-emotional changes after traumatic brain injury. J. Neuropsychol..

[13] Kerr Z.Y., Evenson K.R., Rosamond W.D., Mihalik J.P., Guskiewicz K.M., Marshall S.W. (2014). Association between concussion and mental health in former collegiate athletes. Inj. Epidemiol..

[14] Spinella M. (2007). Normative data and a short form of the Barratt Impulsiveness Scale. Int. J. Neurosci..

[15] Hooper S.R., Alexander J., Moore D., Sasser H.C., Laurent S., King J., Bartel S., Callahan B. (2004). Caregiver reports of common symptoms in children following a traumatic brain injury. Neurorehabilitation.

[16] Zuckerman M. (1994). Behavioral Expressions and Biosocial Bases of Sensation Seeking.

[17] Sharma L., Markon K.E., Clark L.A. (2014). Toward a theory of distinct types of “impulsive” behaviors: A meta-analysis of self-report and behavioral measures. Psychol. Bull..

[18] Smith G.T., Fischer S., Cyders M.A., Annus A.M., Spillane N.S., McCarthy D.M. (2007). On the validity and utility of discriminating among impulsivity-like traits. Assessment.

[19] Dretsch M.N., Silverberg N., Gardner A.J., Panenka W.J., Emmerich T., Crynen G., Ait-Ghezala G., Chaytow H., Mathura V.S., Crawford F. (2017). Genetics and other risk factors for past concussions in active-duty soldiers. J. Neurotrauma.

[20] Hollis S.J., Stevenson M.R., McIntosh A.S., Shores E.A., Collins M.W., Taylor C.B. (2009). Incidence, risk, and protective factor of mild traumatic brain injury in a cohort of Australian nonprofessional male rugby players. Am. J. Sports. Med..

[21] Kim E. (2002). Agitation, aggression, and disinhibition syndromes after traumatic brain injury. NeuroRehabilitation.

[22] O’Connor K.L., Allred C.D., Cameron K.L., Campbell D.E., Houston M.N., Johnson B.R., D’Lauro C., McGinty G., O’Donnell P.G., Peck K.Y. (2017). The prevalence of concussion within the military academies: Findings from the Concussion Assessment, Research, and Education (CARE) Consortium. Br. J. Sports Med..

[23] Veliz P., McCabe S.E., Eckner J.T., Schulenberg J.E. (2019). Concussion, sensation-seeking and substance use among US adolescents. Subst. Abus..

[24] Schroth M.L. (1995). A comparison of sensation seeking among different groups of athletes and nonathletes. Personal. Individ. Differ..

[25] Allen M.S., Magee C.A., Vella S.A., Laborde S. (2017). Bidirectional association between personality and physical activity in adulthood. Health Psychol..

[26] Caspi A., Begg D., Dickson N., Harrington H., Langley J., Moffitt T.E., Silva P.A. (1997). Personality differences predict health-risk behaviors in young adulthood: Evidence from a longitudinal study. J. Personal. Soc. Psychol..

[27] Beidler E., Donnellan M.B., Covassin T., Phelps A.L., Kontos A.P. (2017). The association between personality traits and sport-related concussion history in collegiate student-athletes. Sport Exerc. Perform. Psychol..

[28] Garden N., Sullivan K., Lange R. (2010). The relationship between personality characteristics and postconcussion symptoms in a nonclinical sample. Neuropsychology.

[29] Merritt V.C., Rabinowitz A.R., Arnett P.A. (2015). Personality factors and symptom reporting at baseline in collegiate athletes. Dev. Neuropsychol..

[30] Rush B.K., Malec J.F., Moessner A.M., Brown A.W. (2004). Preinjury personality traits and the prediction of early neurobehavioral symptoms following mild traumatic brain injury. Rehabil. Psychol..

[31] Cross C.P., Cyrenne D.M., Brown G.R. (2013). Sex differences in sensation-seeking: A meta-analysis. Sci. Rep..

[32] Glicksohn J., Naor-Ziv R., Leshem R., Martel M.M. (2018). Sensation seeking and risk-taking. Developmental Pathways to Disruptive, Impulse-Control, and Conduct Disorder.

[33] Shulman E.P., Harden K.P., Chein J.M., Steinberg L. (2015). Sex differences in the developmental trajectories of impulse control and sensation-seeking from early adolescence to early adulthood. J. Youth Adolesc..

[34] Ozer D.J., Benet-Martinez V. (2006). Personality and the prediction of consequential outcomes. Annu. Rev. Psychol..

[35] Hoyle R.H., Stephensen M.T., Palmgreen P., Lorch E.P., Donohew R.L. (2002). Reliability and validity of a brief measure of sensation seeking. Personal. Individ. Differ..

[36] Jack S.J., Ronan K.R. (1998). Sensation seeking among high- and low-risk sports participants. Personal. Individ. Differ..

[37] Castanier C., Le Scanff C., Woodman T. (2010). Who takes risks in high-risks sports? A typological personality approach. Res. Q. Exerc. Sport.

[38] Lynne-Landsman S.D., Graber J.A., Nichols T.R., Botvin G.J. (2011). Is sensation seeking a stable trait or does it change over time?. J. Youth Adolesc..

[39] Zuckerman M. (2007). Sensation Seeking and Risky Behavior.

[40] Alosco M.L., Fedor A.F., Gunstad J. (2014). Attention deficit hyperactivity disorder as a risk factor for concussion in NCAA division-I athletes. Brain Inj..

[41] Iverson G.L., Wojtowicz M., Brooks B.L., Maxwell B.A., Atkins J.E., Zafonte R., Berkner P.D. (2020). High school athletes with ADHD and learning difficulties have a greater lifetime concussion history. J. Atten. Disord..

[42] Nelson L.D., Guskiewicz K.M., Marshall S.W., Hammeke T., Barr W., Randolph C., McCrea M.A. (2016). Multiple self-reported concussions are more prevalent in athletes with ADHD and learning disability. Clin. J. Sport Med..

[43] Kerr Z.Y., Register-Mihalik J.K., Kroshus E., Baugh C.M., Marshall S.W. (2015). Motivations associated with nondisclosure of self-reported concussions in former collegiate athletes. Am. J. Sports Med..

[44] LaRoche A.A., Nelson L.D., Connelly P.K., Walter K.D., McCrea M.A. (2016). Sport-related concussion reporting and state legislative effects. Clin. J. Sports Med..

[45] Broglio S.P., McCrea M., McAllister T., Harezlak J., Katz B., Hack D., Hainline B., CARE Consortium Investigators (2017). A national study on the effects of concussion in collegiate athletes and US military service academy members: The NCAA-DoD Concussion Assessment, Research and Education (CARE) Consortium structure and methods. Sports Med..

[46] Broglio S.P., Kontos A.P., Levin H., Schneider K., Wilde E.A., Cantu R.C., Feddermann-Demont N., Fuller G.W., Gagnon I., Gioia G.A. (2018). National Institute of Neurological Disorders and Stroke and Department of Defense sport-related concussion common data elements version 1.0 recommendations. J. Neurotrauma.

[47] Carney N., Ghajar J., Jagoda A., Bedrick S., Davis-O’Reilly C., du Coudray H., Hack D., Helfand N., Huddleston A., Nettleton T. (2014). Concussion guidelines step 1: Systematic review of prevalent indicators. Neurosurgery.

[48] Stephenson M.T., Hoyle R.H., Palmgreen P., Slater M.D. (2003). Brief measures of sensation seeking for screening and large-scale surveys. Drug Alcohol Depend..

[49] Rice S.G., American Academy of Pediatrics Council on Sports Medicine and Fitness (2008). Medical conditions affecting sports participation. Pediatrics.

[50] Saletti S.R., Chang D.O., Pérez-Aranibar C.C., Campos F.O. (2017). Psychometric properties of the Brief Sensation Seeking Scale in Peruvian teenagers. Psicothema.

[51] Primi C., Narducci R., Benedetti D., Donati M., Chiesi F. (2011). Validity and reliability of the Italian version of the Brief Sensation Seeking Scale (BSSS) and its invariance across age and gender. Test. Psychom. Methodol. Appl. Psychol..

[52] Caccese J.B., DeWolf R.M., Kaminski T.W., Broglio S.P., McAllister T.W., McCrea M., Buckley T.A. (2019). Estimated age of first exposure to American football and neurocognitive performance amongst NCAA male student-athletes: A cohort study. Sports Med..

